# Stem Cell Mobilization with Ixazomib and G-CSF in Patients with Multiple Myeloma

**DOI:** 10.3390/cancers15020430

**Published:** 2023-01-09

**Authors:** Selina Bühler, Dilara Akhoundova, Barbara Jeker, Myriam Legros, Katja Seipel, Michael Daskalakis, Ulrike Bacher, Thomas Pabst

**Affiliations:** 1 Department of Medical Oncology, Inselspital, Bern University Hospital, University of Bern, 3010 Bern, Switzerland; 2 Department of Hematology and Central Hematology Laboratory, Inselspital, Bern University Hospital, University of Bern, 3010 Bern, Switzerland; 3 Department of Biomedical Research, University of Bern, 3008 Bern, Switzerland

**Keywords:** multiple myeloma, stem cell mobilization, autologous stem cell transplantation (ASCT), ixazomib

## Abstract

**Simple Summary:**

High-dose chemotherapy (HDCT) followed by autologous stem cell transplantation (ASCT) is the standard consolidation strategy for patients with newly diagnosed multiple myeloma (MM), as well as for a subset of patients with relapsed/refractory disease. ASCT reduces the duration of myelosuppression induced by HDCT. Chemotherapy agents combined with granulocyte-colony stimulating factors (G-CSF), as well as plerixafor, are key components of currently used stem cell mobilization regimens. However, chemotherapy mobilizing agents are associated with risk of infectious complications and peripheral neuropathy, which is as well a common toxicity of many drugs used in MM treatment. Ixazomib is an oral proteasome inhibitor and has lower neurotoxic potential. We for the first time combined ixazomib with G-CSF (filgrastim) for stem cell mobilization in patients with MM undergoing HDCT and ASCT, and assessed safety and efficacy of this mobilization strategy. Ixazomib was globally well tolerated and no new toxicities have been observed. The combination of ixazomib and G-CSF showed promising stem cell mobilizing activity and led to successful stem cell mobilization in 17 out of 19 (89%) patients. However, 9 (47%) patients required the addition of plerixafor to ensure optimal stem cell collection. Future larger studies might further investigate the role of ixazomib in stem cell mobilization regimens for MM.

**Abstract:**

(1) Background: High-dose chemotherapy (HDCT) followed by autologous stem cell transplantation (ASCT) is the standard consolidation strategy for patients with newly diagnosed multiple myeloma (MM) and for a subset of patients with relapsed/refractory disease. For stem cell mobilization, G-CSF alone or in combination with chemotherapy mobilizing agents and/or plerixafor are commonly used. Ixazomib is an oral proteasome inhibitor with less neurotoxic potential, which previously showed the ability to mobilize stem cells in preclinical studies. (2) Methods: Prospective single-center phase 1 study assessing the efficacy and safety of stem cell mobilization with ixazomib and G-CSF in patients with newly diagnosed or relapsed/refractory MM undergoing HDCT and ASCT. Primary endpoint was percentage of patients achieving a yield of at least 6.0 × 10^6^/kg CD34+ cells within the first apheresis. G-CSF (filgrastim) 10 μg/kg/day was administered subcutaneously (s.c.) from day 1 to day 5 (planned apheresis) and ixazomib 4 mg orally at day 4. Plerixafor 24 mg s.c. was administered if the stem cell mobilization with ixazomib and G-CSF was not sufficient. (3) Results: 19 patients were treated within the study between 06/2020 and 02/2021. The primary endpoint was reached in 17 (89%) patients, with a median of 7.1 × 10^6^/kg CD34+ cells collected within the first apheresis, comparable to previously published results, and only 2 (11%) patients required a second apheresis. Median number of circulating CD34+ cells was 14.0 × 10^6^/L (2.0–95.2) before the administration of ixazomib, and 33.0 × 10^6^/L (4.2–177.0) pre-apheresis. However, 9 (47%) patients required the addition of plerixafor to ensure optimal stem cell collection. (4) Conclusions: The combination of ixazomib and G-CSF showed promising stem cell mobilizing activity in patients with MM prior to HDCT and ASCT. Future larger studies might further investigate the role of ixazomib in stem cell mobilization regimens for MM.

## 1. Introduction

Multiple myeloma (MM) is a hematologic malignancy characterized by neoplastic proliferation of plasma cells, which generate large amounts of circulating monoclonal immunoglobulin and/or light chains. This plasma cell proliferation occurs in the bone marrow, often leading to osteolytic bone lesions. Other common disease manifestations of MM are anemia, hypercalcemia and renal insufficiency [1,2]. Following induction therapy, high-dose chemotherapy (HDCT) and autologous stem cell transplantation (ASCT) is the standard consolidation strategy for younger adults and selected fit elderly patients with newly diagnosed MM, and a subset of patients with relapsed or refractory MM [3,4,5,6,7,8]. However, HDCT regimens lead to severe and prolonged myelosuppression. To reduce the duration of myelosuppression and the associated morbidity and mortality, transplantation of autologous hematopoietic stem cells is required following HDCT [9,10]. Therefore, a previous successful stem cell mobilization and collection are essential [5,11]. Physiologically, hematopoietic stem cells are located in the bone marrow and only a small amount circulates in peripheral blood. Through stimulation with cytokines and/or chemotherapy, the number of circulating stem cells can be significantly increased [12]. Several factors, such as patient’s age, the induction treatment regimen and bone marrow disease extension, relevantly impact the mobilization efficiency [13]. The minimum amount of CD34+ cells required for a successful ASCT is 2.0 × 10^6^ cells/kg [14,15]. For stem cell mobilization, granulocyte colony-stimulating factors (G-CSF) are most frequently used. G-CSF alone lead to suboptimal stem cell mobilizations in 5–30% of patients. Therefore, they are commonly combined with chemotherapeutic drugs such as vinorelbine, cyclophosphamide or gemcitabine [16,17,18,19,20]. Another active drug used for stem cell mobilization is plerixafor, a selective and reversible CXCR4 inhibitor. Plerixafor has synergistic activity with G-CSF and results in low rates of mobilization failure [21,22,23,24]. In Switzerland, the standard regimen for stem cell mobilization is a combination of vinorelbine and G-CSF. Some advantages of vinorelbine, as compared to cyclophosphamide, are the good predictability of the stem cell collection at day 8, the feasibility of outpatient management and a lower rate of infectious complications [6,25,26]. 

The neurotoxicity of vinorelbine however is a major handicap since a relevant proportion of MM patients have preexistent peripheral neuropathy, partly due to neurotoxic drugs used within induction regimens. It has been shown that the use of vinorelbine in these patients can aggravate this preexisting chemotherapy-induced peripheral neuropathy (CIPN) [27]. The occurrence of CIPN can relevantly limit subsequent therapeutic options. Moreover, CIPN is an important cause of morbidity, with relevant negative impact on patient’s quality of life [28]. Thus, development of less toxic mobilizing regimens remains a relevant unmet clinical need. 

Ixazomib is an oral, highly selective and reversible proteasome inhibitor characterized by low neurotoxicity [29,30]. The combination of ixazomib with lenalidomide and dexamethasone prolonged PFS in patients with relapsed or refractory MM, as compared to lenalidomide and dexamethasone alone [31,32]. In mice, single-dose ixazomib could successfully mobilize hematopoietic stem and progenitor cells, and improved the mobilization effect of G-CSF [33]. Bortezomib, another proteasome inhibitor, has demonstrated stem cell mobilizing activity, enhancing in vitro mobilization efficacy of G-CSF. Downregulation of stem cell adhesion proteins, as well as modification of bone marrow cytokine levels have been proposed as possible mechanisms of action [34,35]. For ixazomib, similar mechanisms of action have been postulated, although mechanistic studies are still lacking [33]. Based on this preclinical efficacy data and favorable toxicity profile, we hypothesized that the addition of ixazomib to G-CSF could be an attractive strategy for stem cell mobilization in patients with MM.

In this phase 1 study, we assessed the mobilization efficacy and safety of ixazomib in combination with G-CSF in patients with MM undergoing HDCT followed by ASCT. We additionally monitored the effect of ixazomib alone on circulating levels of CD34+ cells in two patients undergoing maintenance therapy with ixazomib. This is the first study investigating the role of ixazomib in stem cell mobilization in MM patients.

## 2. Materials and Methods

### 2.1. Study Design and Participants

19 patients with MM undergoing HDCT and ASCT at the University Hospital of Bern (Inselspital) between June 2020 and February 2021 were included in this prospective phase 1 study. Patients with at least partial response (PR) after induction treatment were eligible. Moreover, we monitored CD34+ cell levels in two additional patients receiving a maintenance therapy with ixazomib. All patients provided written informed consent and the study was approved by the local ethics committee of Bern, Switzerland (decision number #2018-00615).

### 2.2. Outcomes

Primary endpoint of the study was percentage of patients achieving a yield of at least 6.0 × 10^6^/kg CD34+ cells within the first apheresis. Secondary endpoints were number of circulating CD34+ cells measured before apheresis, number of apheresis required, percentage of patients requiring use of plerixafor, safety of ixazomib and G-CSF, as wells as hematologic recovery and infectious complications after HDCT and ASCT.

### 2.3. Procedures

#### 2.3.1. Stem Cell Mobilization and HDCT Regimens

All 19 patients included in the study received ixazomib in combination with G-CSF (filgrastim) for stem cell mobilization. Filgrastim was administered subcutaneously (sc) following a weight adapted dosing (patients with up to 69 kg body weight received 60 million international units (MIU), from 70 kg to 88 kg, 78 MIU, and over 89 kg, 96 MIU) from day 1 to the day of the apheresis (planed on day 5). Ixazomib 4 mg was administered as a single-dose orally at day 4, within an interval of 12 to 24 h before apheresis. Patients with insufficient stem cell mobilization received additionally plerixafor 24 mg sc, 8–10 h prior to apheresis. Apheresis for stem cell collection was performed between day 4 and 6. The target value of collected CD34^+^ cells was defined as 6 × 10^6^/kg. 

Following successful stem cell collection, patients underwent HDCT, receiving one of the following two regiments: high-dose treosulfan and melphalan (TreMel) or split-dose melphalan, depending on patient age and comorbidities. The TreMel regimen consisted of intravenous (iv) treosulfan 14 g/m^2^/day on days −4 to −2 before ASCT, combined with melphalan 140 mg/m^2^ iv on day −1. Patients who were treated with melphalan 200 mg/m^2^, received a split-dose schedule (100 mg/m^2^/day) at day −2 and −1 before ASCT.

#### 2.3.2. Supportive Therapy

During stem cell mobilization all patients received meloxicam to enhance stem cell mobilization and ondansetron as prophylactic antiemetic therapy. Sulfamethoxazole-trimethoprim was given as prophylaxis for pneumocystis jiroveci infection from the start of HDCT until 3 weeks after ASCT. Fluconazol was administered from HDCT until the end of aplasia to prevent fungal infections, and valaciclovir as virostatic prophylaxis from day +1 until 3 months post-ASCT. Dexamethasone was given from day −4 to ASCT and from day +9 to +13 after ASCT as prophylaxis of engraftment syndrome. Antiallergic prophylaxis before ASCT was performed with iv methylprednisolone and clemastine. Allopurinol was given to prevent tumor lysis syndrome during HDCT. Additionally, after ASCT all patients received folic acid for 8 weeks to improve hematopoietic recovery. Zoledronic acid was given at day +1 after ASCT. All patients received G-CSF (filgrastim) 5 μg/kg/day from day +6 to +12 after ASCT. Further supportive medication included aprepitant, ondansetron, esomeprazole, enoxaparin natrium and furosemide. 

#### 2.3.3. Blood Samples Collection and Analysis

To analyze the effect of ixazomib alone (without G-CSF) on the mobilization of hematopoietic stem cells, we measured the levels of leukocytes and CD34^+^ cells in patients receiving a maintenance therapy with ixazomib. We assessed these values before, 9–10 h, 13 h and 16–17 h after the administration of ixazomib. To analyze the effect of ixazomib combined with G-CSF on hematopoietic mobilization, we measured the circulating CD34+ cells in all 19 patients included in the phase1 study before ixazomib administration, at day 5 of mobilization and before apheresis. Additionally, neutrophils counts were measured at baseline, before ixazomib administration, at day 5 of mobilization and before apheresis. In one patient we additionally monitored the CD34+ and leukocytes cell count at 3 h, 8 h, 12 h, 16 h and 19 h after the administration of ixazomib in combination with G-CSF. 

### 2.4. Response Assessment

Treatment response to induction regimens and HDCT was assessed following the International Myeloma Working Group (IMWG) uniform response criteria for MM [36]. Complete response (CR) was defined as negative serum and urine immunofixation, radiographical absence of plasmacytoma and less than 5% plasma cells in the bone marrow. Stringent complete response (sCR) was defined as CR with MRD-negativity (<10^−5^). PR was defined as at least a 50% reduction of serum M-protein. Very good partial response (VGPR) was defined as a 90% reduction of serum M-protein and urine M-protein <100 mg/24 h, or serum and urine M-protein only detectable by immunofixation but not on electrophoresis [37]. Progressive disease (PD) was defined as increase of monoclonal immunoglobulins in serum or urine of at least 25% or an increase of light chains in urine of 25%. If the criteria for CR, VGPR, PR and PD were not fulfilled, this was defined as stable disease (SD).

### 2.5. Statistical Analysis

Descriptive analysis (e.g., median, range) for categorical variables was performed. Analyses, tables and graphs have been performed using Microsoft Excel version 16.65. Data collection cut-off date was 1 July 2022. 

## 3. Results

### 3.1. Patient Characteristics 

Patient characteristics at initial diagnosis of multiple MM are summarized in Table 1. 

### 3.2. Multiple Myeloma Treatment before Stem Cell Mobilization

16 (84%) patients received HDCT and ASCT for newly diagnosed MM, and 3 (16%) patients for relapsed/refractory disease. 13 patients received an induction therapy with lenalidomide, bortezomib and dexamethasone (VRd) and 4 patients received a different regimen (Table 2). The median number of treatment cycles before stem cell mobilization was 4 (range 3–6). Seven patients additionally received radiation to symptomatic bone lesions. After induction therapy, 16% of patients achieved CR, 32% a VGPR, 42% a PR, and 11% a SD. No PD was observed. Details of treatment regimens and responses preceding stem cell mobilization are summarized in Table 2.

### 3.3. Stem Cell Mobilizing Efficacy of Ixazomib without G-CSF

**Figure 1:**
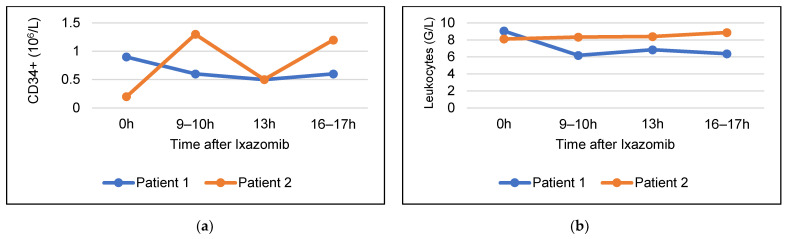
Modifications in (**a**) CD34+ cells and (**b**) leukocytes levels in two patients following administration of ixazomib.

The CD34+ cells and leukocytes counts of two patients undergoing maintenance therapy with ixazomib were monitored following the administration of ixazomib. In patient 1 blood count was performed 10 h, 13 h and 16 h after ixazomib, and in patient 2, 9 h, 13 h and 17 h after ixazomib. Figure 1 illustrates the modifications in the CD34+ cells and leukocytes levels in these two patients. In patient #1 no increase in CD34+ cells or leukocytes was observed. The initial value of the CD34+ cells was 0.9 × 10^6^/L, decreasing to 0.6 × 10^6^/L 17 h post-ixazomib, and the leukocytes decreased from 9.06 G/L to 6.38 G/L. In patient #2 the number of CD34+ cells increased 6.5 times from baseline after 9 h (from 0.2 × 10^6^/L to 1.3 × 10^6^/L), showing a decrease to 0.5 × 10^6^/L at 13 h, and a new increase to 1.2 × 10^6^/L after 17 h. No relevant variation in the leukocytes count was observed.

### 3.4. Stem Cell Mobilization with Ixazomib and G-CSF

**Figure 2:**
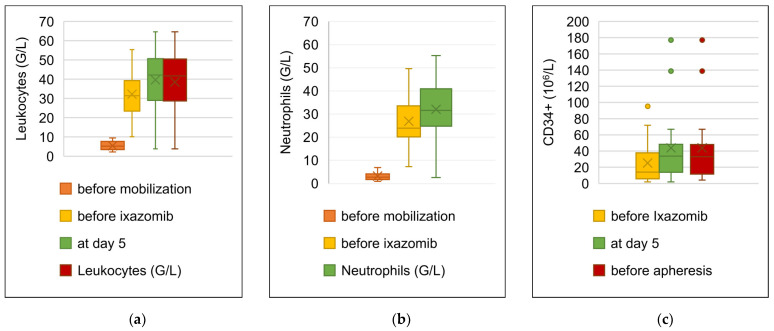
Box plot illustrating modifications in number of circulating (**a**) leukocytes, (**b**) neutrophils and (**c**) CD34+ cells during stem cell mobilization with ixazomib and G-CSF.

All 19 patients received G-CSF (filgrastim) 10 μg/kg/day sc starting from day 1 to day 5 (planned apheresis). 17 patients received a single dose of ixazomib 4 mg on day 4, one patient on day 3 and one patient on day 5. The median number of leukocytes before mobilization was 5.2 G/L (2.3–9.4), 31.4 G/L (10.1–55.4) before the administration of ixazomib, and 42.2 G/L (3.8–64.6) at day 5. The median number of leukocytes before stem cell collection was 41.7 G/L (3.84–64.6). The circulating number of neutrophils increased from the initial median value of 2.7 G/L (0.9–6.9) to 23.9 G/L (49.6–7.3) before the administration of ixazomib and up to 31.6 G/L (2.6–55.2) at day 5. The median number of CD34+ cells at day 4 was 14.0 × 10^6^/L (2.0–95.2), increasing to 33.7 × 10^6^/L (1.8–177) at day 5, and 33.0 × 10^6^/L (4.2–177.0) before apheresis. Figure 2 illustrates the modifications in number of circulating cells during stem cell mobilization.

**Figure 3:**
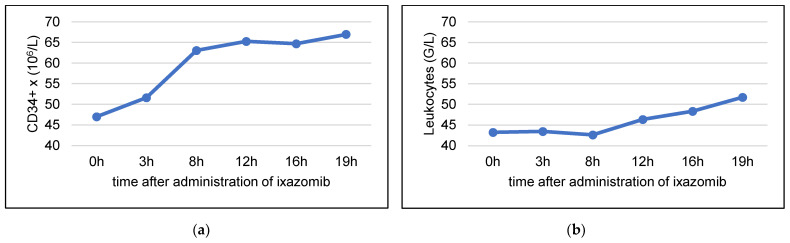
Serial assessment of (**a**) peripheral CD34+ cells and (**b**) leukocytes following administration of ixazomib (single-dose) and GCSF.

In one patient, we additionally performed serial monitoring of circulating CD34+ cells and leukocytes at 3 h, 8 h, 12 h, 16 h and 19 h after ixazomib intake (Figure 3). The fastest increase of CD34+ cells was observed between 3 and 8 h after ixazomib administration, and peak values were detected at 19 h.

### 3.5. Results of Stem Cell Apheresis

Study primary endpoint was reached for 17 out of 19 (89%) patients, and the median number of collected CD34+ cells was 7.1 × 10^6^/kg body weight. Regarding secondary endpoints, median number of CD34+ cells before apheresis was 33.0 (4.2–177.0) × 10^6^/kg; 2 (11%) patients required an additional apheresis to allow sufficient stem cell collection, and plerixafor was used in 9 (47%) patients to allow optimal stem cell collection. 2 out of this 9 patients had r/r multiple myeloma. Stem cell apheresis could be performed as planned on day 5 in 14 patients, on day 6 in 3 patients, and on day 4 in 2. The apheresis took place one day after ixazomib administration in 16 patients, in 2 patients the apheresis was performed two days after ixazomib administration, and one patient had the apheresis on the same day. The median duration of the apheresis was 313 min and the median processed blood volume was 28,506 mL. Further details related to stem cell mobilization and apheresis are summarized in Table 3.

### 3.6. Safety of the Mobilization with Ixazomib and G-CSF

Single-dose ixazomib was well tolerated, and no unexpected adverse events occurred. No new or increase in preexisting peripheral neuropathy symptoms were registered, and no infectious complications occurred during stem cell mobilization and previously to HDCT and ASCT.

### 3.7. High-Dose Chemotherapy and Autologous Stem Cell Transplantation

17 patients were treated with the treosulfan/melphalan (TreMel) conditioning regimen, two patients received melphalan monotherapy. Median time from start of mobilization to autologous stem cell transplantation was 15 days (range: 12–49) and the median number of transplanted CD34+ cells was 3.2 × 10^6^/kg (1.5–6.7 × 10^6^/kg).

### 3.8. Hematologic Recovery after HDCT-ASCT and Infectious Complications

Details of hematologic recovery and infectious complications are summarized in Table 4. Median duration of hospitalization was 21 days. Median time to recovery of platelets to ≥20 G/L and neutrophils to ≥0.5 G/L was 14 and 12 days, respectively. 17 (89%) patients needed platelet transfusions (median of 2 concentrates) and 12 (63%) patients required erythrocyte transfusions (median of 1 concentrate). At least one febrile episode occurred in all patients. In 32% of the cases a bacterial pathogen was identified (including 3 patients with positive blood cultures), and in 5%, a viral pathogen. In 63% of the patients, no positive microbiological findings could be identified.

One patient had an infection of the upper airways caused by rhinoviruses/enteroviruses and another patient had a SARS-CoV-2 (COVID-19) infection leading to an acute respiratory distress syndrome. One patient had a septic thrombosis of the V. jugularis following a catheter associated infection. Engraftment syndrome occurred in one patient. The same patient also developed a soft tissue abscess, where no pathogen could be identified. 15 (79%) patients did not have relevant organ toxicities or infectious complications.

### 3.9. Remission Status after HDCT and ASCT

Following treatment consolidation with HDCT and ASCT, 42% of patients achieved a sCR, 21% a CR, 11% a VGPR, 21% a PR, and 5% a SD. No disease progression was observed in the first bone marrow biopsy following HDCT. At cut-off date, 1 July 2022, 7 patients had a progression of their disease and 3 patients died from MM, with a median survival time of 12 months after the transplantation for these patients. Outcome data are summarized in Table 5.

## 4. Discussion

To date there is a lack of consensus as to the optimal stem cell mobilization regimen for patients with MM undergoing treatment consolidation with HDCT and ASCT, and a broad spectrum of mobilization protocols is used across different institutions [38,39]. G-CSF, cytostatic chemotherapy agents and plerixafor are key components of these protocols [11,12]. While G-CSF alone can successfully mobilize stem cells into peripheral blood, the addition of chemotherapy agents improved mobilization efficacy, especially in more heavily pre-treated patients [40,41,42,43]. However, a relevant limitation of chemotherapy-based mobilization regimens is the high rate of related adverse event, such as cytopenia, febrile neutropenia, infectious complications and neurotoxicity [19,27]. While cyclophosphamide has been most frequently used in MM mobilization regimens [38], in Switzerland the standard regimen for stem cell mobilization is the combination of vinorelbine and G-CSF. As compared to cyclophosphamide, which has been classically used in several MM mobilization studies, vinorelbine is associated with lower rates of adverse events and infectious complications and can be easily administered in the outpatient setting. Moreover, stem cell mobilization with vinorelbine leads to a predictable CD34+ cell peak at day 8 [6,25,26]. Still, vinorelbine entails a relevant risk of neurotoxicity [27].

Based on preclinical efficacy data of ixazomib [33] and given it’s favorable toxicity profile [29], we aimed to evaluate the stem cell mobilizing efficacy of G-CSF combined with ixazomib in MM patients undergoing HDCT and ASCT. In order to assess the effect of ixazomib on circulating CD34+ cell levels, we performed serial monitoring of CD34+ cells at baseline and following ixazomib administration in two patients receiving ixazomib maintenance. In one of these patients, a moderate increase in circulating CD34+ cells was observed, with a peak occurring 9 h post-ixazomib administration. In the other patient, no modifications in the level of CD34+ cells was observed. We concluded that the amount of CD34+ cells mobilized with ixazomib alone would be insufficient to allow successful stem cell collection. Bortezomib and ixazomib have shown synergistic activity with G-CSF in pre-clinical studies [34,35], and single-dose bortezomib, added to a 5 day G-CSF schedule, has been assessed in a phase 1 study, showing peak stem cell mobilization 15 to 18 h following bortezomib administration. The proteasome inhibitor bortezomib has been shown to downregulate the stromal vascular cell adhesion molecule (VCAM-1), modulating the very late activation antigen-4 (VLA-4)/VCAM-1 interaction between the hematopoietic progenitors and the bone marrow stroma [34,44]. A similar mobilization mechanism has been hypothesized for ixazomib [33].

In our phase 1 study we combined single-dose ixazomib with a 5-day weight-adapted G-CSF schedule. In all 19 patients, we were able to collect enough hematopoietic stem cells and the planned transplantation could be successfully conducted. The median number of collected CD34+ cells in the first apheresis was 7.0 × 10^6^/kg. This value is comparable to the amount of hematopoietic stem cells which could be mobilized and collected with the combination of vinorelbine and G-CSF or cyclophosphamide and G-CSF [19,26,45]. Eight MM studies have been included in a network meta-analysis assessing the efficacy of distinct stem cell mobilization regimens across several hematologic malignancies. This meta-analysis showed that the combination of cyclophosphamide and reduced-dose G-CSF (5–7.5 μg/kg/day) lead to increased yield of CD34+ cells, as compared to standard-dose G-CSF (10 μg/kg/day) alone [38]. In one of these studies, cyclophosphamide and G-CSF lead to a CD34+ cells yield of 4.0 (0.8–12.4) vs. 2.7 (0.5–12.4) × 10^6^/kg (*p* = 0.023), respectively [46]. In this study, plerixafor was used in 6% of patients in the combination arm vs. in 14% in the G-CSF monotherapy arm. In our cohort, a higher rate of plerixafor use was required, in 9 (47%) patients, to ensure optimal stem cell mobilization and collection. Plerixafor is a reversible inhibitor of the chemokine receptor CXCR4. By blocking the interaction of CXCR4 with the stromal-cell derived factor-1 (SDF-1), plerixafor impairs the adherence of hematopoietic stem cells within the bone marrow microenvironment [47], improving the collection of peripheral blood stem cells. However, it’s use is primarily restricted to patients with failure of mobilization with standard regimens including G-CSF and/or chemotherapy [13]. Only two patients (the first one with r/r multiple myeloma, the second one with newly diagnosed MM) additionally required a second apheresis to collect the target amount of CD34+ cells. Overall single-dose ixazomib was well tolerated, and no unexpected adverse events occurred.

Our study does have some limitations. The sample size is relatively small and includes a heterogeneous population, since both newly diagnosed and relapsed/refractory MM patients could be included. Moreover, patients received distinct induction regimens, with potential impact on mobilization efficacy. Finally, the study lacks comparison between stem cell mobilization with G-CSF alone and G-CSF combined with ixazomib. Despite these limitations, our study demonstrates, to our knowledge for the first time, the feasibility of stem cell mobilization with the combination of ixazomib and G-CSF in patients with MM and provides promising efficacy data.

## 5. Conclusions

This phase 1 study assessed for the first time the efficacy and safety of ixazomib in combination with G-CSF for stem cell mobilization in patients with MM undergoing treatment consolidation with HDCT and ASCT. Stem cell mobilization with ixazomib and G-CSF was well tolerated, and no neurotoxic complication were observed. Despite high rate of plerixafor use, the combination of ixazomib and G-CSF showed promising activity, leading to successful stem cell mobilization in 89% of the patients. Future larger studies might further investigate the role of ixazomib within MM stem cell mobilization regimens.

## Figures and Tables

**Table 1:** Patient characteristics at diagnosis.

Parameter	Results
Number of patients, n	19
Median age, years, n (range)	65 (38–75)
Sex: male/female, n (%)	15 (79%)/4 (21%)
Heavy chain subtype, n (%)	
IgG ^a^	14 (74%)
IgA	4 (21%)
IgM	0 (0%)
Light chain only Light chain subtype	1 (5%)
Lambda	7 (37%)
Kappa	12 (63%)
Organ involvement Confirmed amyloidosis, n (%) Hypercalcemia (>2.6 mmol/L), n (%) Renal insufficiency (eGFR ^b^ <60 mL/min), n (%) Creatinine (µmol/L), median (range) Presence of osteolytic lesions, n (%)	0 (0%) 2 (11%) 3 (16%) 82 (53–322) 16 (84%)
Anemia (hemoglobin < 110 g/L), n (%)	12 (63%)
Hemoglobin, g/L, median (range) ^c^	97.0 (103.0–139.0)
Cytogenetic alterations, n (%)	
High-risk ^d^	6 (32%)
Standard-risk	13 (68%)
β-2-Microglobulin (mg/L), median (range)	3.36 (1.92–21)
Albumin (g/L), median (range)	35 (20–44)
Stage, R-ISS ^e^, n (%)	
I	4 (21%)
II	8 (42%)
III	7 (37%)

^a^ Ig: Immunoglobulin, ^b^ eGFR: estimated glomerular filtration rate, ^c^ no data available from 2 patients. ^d^ High-risk: deletion 17p; translocations t(4;14), t(14;16) and t(14;20); +1q (2 patients) and *TP53* mutation. ^e^ R-ISS: Revised International Staging System.

**Table 2:** Details of induction treatment regimens and responses before stem cell mobilization.

Parameter	Results
Patients with newly diagnosed MM, n (%) Patients with relapsed/refractory MM, n (%)	
Induction regimen, n (%)	
VRd ^a^	15 (79%)
VD ^b^	1 (5%)
Bortezomib, Thalidomide, Dexamethasone	1 (5%)
VD (3 cycles), Pegylated Liposomal Doxorubicin, Bortezomib, Dexamethasone (2 cycles)	1 (5%)
VRd (2 cycles), VCd ^c^ (3 cycles)	1 (5%)
Number of cycles, median (range)	4 (3–6)
Symptomatic radiotherapy, n (%)	
Yes	7 (37%)
No	12 (63%)
Remission status previous to HDCT and ASCT, n (%)	
Stringent complete response	0 (0%)
Complete response	3 (16%)
Very good partial response	6 (32%)
Partial response	8 (42%)
Stable disease	2 (11%)
Progressive disease	0 (0%)

^a^ VRd: Velcade, Revlimid, Dexamethasone; ^b^ VD: Velcade, Dexamethasone; ^c^ VCd: Velcade, Cyclophosphamide, Dexamethasone.

**Table 3:** Characteristics and results of stem cell mobilization and apheresis.

Parameter	Results
**Stem Cell Mobilization**	
Median age, years (range)	65 (38–75)
Duration of G-CSF ^a^ administration before apheresis, days, median (range)	5 (4–6)
Time interval from ixazomib administration to apheresis, days, median (range)	1 (0–2)
Plerixafor use, n (%)	
Yes	9 (47%)
No	10 (53%)
Leukocytes count, G/L, median (range)	
before mobilization	5.2 (2.3–9.5)
before ixazomib ^b^	31.4 (10.1–55.4)
at day 5 before apheresis	42.2 (3.8–64.6) 41.7 (3.8–64.6)
Neutrophil granulocytes count, G/L, median (range)	
before mobilization	2.7 (0.9–6.9)
before ixazomib ^b^	23.9 (49.6–7.3)
at day 5 ^c^	31.6 (2.6–55.2)
CD34^+^ cells count, 10^6^/L, median (range)	
before ixazomib ^d^	14.0 (2.0–95.2)
at day 5	33.7 (1.8–177.0)
before apheresis	33.0 (4.2–177.0)
**Stem cell apheresis**	
Duration of apheresis, minutes, median (range)	313 (144–438)
Processed blood volume, ml, median (range)	28,506 (15,387–57,489)
Number of collected CD34^+^ cells, 10^6^/kg body weight, median (range)	7.1 (2.9–21.6)
Second apheresis, n (%)	2 (11%)

^a^ G-CSF: granulocyte-colony stimulating factor, ^b^ no data from 3 patients. ^c^ No data available from 6 patients. ^d^ No data available from 5 patients.

**Table 4:** Hematologic recovery after HDCT ^a^ and ASCT ^b^ in the study cohort.

Duration of Hospitalization and Time to Hematologic Recovery	
Duration of hospitalization, days, median (range)	21 (17–48)
Time to platelets recovery ≥ 20 G/L, days, median (range)	14 (10–70)
Time to neutrophil granulocytes recovery ≥ 0.5 G/L, days, median (range)	12 (10–16)
Patient requiring platelet transfusions, n (%)	17 (89%)
Number of platelet concentrates, median (range)	2 (0–13)
Patient requiring erythrocyte transfusion, n (%)	12 (63%)
Number of erythrocyte concentrates, median (range)	1 (0–8)
**Infectious complications**	
Febrile episodes, n (%)	19 (100%)
Number of febrile episodes, median (range)	1 (1–2)
Detected pathogen, n (%)	
Viral	1 (5%)
Bacterial	6 (32%)
Fungi	0 (0%)
No pathogen detected	12 (63%)
Positive blood cultures, n (%)	
Yes	3 (16%)
No	16 (84%)

^a^ HDCT: high-dose chemotherapy; ASCT ^b^ autologous stem cell transplantation.

**Table 5:** Follow-up data after HDCT and ASCT in the study cohort.

Remission status after HDCT ^a^, n (%)	
Stringent complete response	8 (42%)
Complete response	4 (21%)
Very good partial response	2 (11%)
Partial response	4 (21%)
Stable disease	1 (5%)
Progressive disease	0 (0%)
Maintenance therapy, n (%)	
Yes	14 (74%)
No	5 (26%)
Progression after HDCT and ASCT, n (%)	7 (37%)
Time from transplantation to progression, months, median (range)	9 (1–14)
Time from transplantation to next treatment, months, median (range) ^b^ Number of deaths Time from transplantation to death, months, median (range)	12 (4–15) 3 (16%) 12 (11–14)
Follow-up, months, median (range)	19 (11–24)

^a^ HDCT: high-dose chemotherapy. ^b^ One patient had not yet initiated next treatment line at the cut-off date and from one patient no data was available.

## Data Availability

The data presented in this study are available on request from the corresponding author.
